# Drug-Metabolizing Gene Expression Identity: Comparison Across Liver Tissues and Model Cell Lines

**DOI:** 10.3390/biomedicines13112722

**Published:** 2025-11-06

**Authors:** Viktoriia A. Arzumanian, Ekaterina V. Timofeeva, Olga I. Kiseleva, Ekaterina V. Poverennaya

**Affiliations:** 1 Institute of Biomedical Chemistry, 119121 Moscow, Russia; 2 Department of Bioengineering and Bioinformatics, Institute of Pharmacy, Sechenov First Moscow State Medical University (Sechenov University), 119991 Moscow, Russia; timokaty02@gmail.com

**Keywords:** cell line, liver, primary hepatocyte, transcriptome, drug-metabolizing enzyme genes

## Abstract

**Background:** Human cell lines underpin modern biomedical research, offering reproducibility, standardisation, and unrestricted access to biological material. Among the 1206 human lines documented in the Human Protein Atlas, in vitro systems overcome the ethical and technical constraints of primary tissues. The liver is an organ of intricate structure, diverse physiological roles, and limited in vitro viability. Liver-derived cell lines are increasingly used to address the growing burden of liver disease and to accelerate pharmaceutical development, yet their capacity to replicate native hepatic functions remains uncertain. The mutational profiles and expression patterns of hepatocyte-characteristic genes provide critical benchmarks for their suitability for pharmacology and toxicology. **Methods:** Here, we systematically compare ten widely used hepatic cell lines (HepG2, Huh7, Hep3B, LX-2, HepaRG, HLF, HLE, MHCC97H, SK-Hep1, PLC/PRF/5) with primary hepatocytes and liver tissue, focusing on drug-metabolizing enzyme (DME) gene expression. Beyond literature synthesis, we analysed pre-processed RNA-seq expression data. **Results:** Overall, among the models examined, the HepaRG cell line shows the greatest similarity to liver and primary hepatocytes, most faithfully reproducing the expression patterns of DME genes. HepG2, Hep3B, and Huh7 form a cluster that retains only a subset of hepatic characteristics. Other models display more pronounced deviations from the reference profile and are generally used for specialized applications. Thus, no universal cell line exists that can fully substitute for the liver. Each model has its own limitations and biases in the expression profile of DME genes, which must be carefully considered when selecting an appropriate system for specific research objectives.

## 1. Introduction

The liver is the body’s primary metabolic organ, orchestrating the processing, storage, and transformation of nutrients, drugs, and toxins. Its metabolic functions are essential for maintaining energy balance, detoxification, and overall homeostasis. The complex architecture and broad physiological repertoire make the liver both vital and vulnerable. Understanding liver biology and pathology requires experimental models that can faithfully recapitulate its cellular and molecular complexity.

For toxicological and pharmaceutical studies, human liver biopsies remain the gold standard for physiological relevance. Yet, their use is hampered by a rapid decline in key metabolic activities, limited sample availability, and donor variability [1]. Cell lines, like primary hepatocytes, are widely used in vitro to study drug metabolism and assess hepatotoxicity, but they share similar limitations in terms of production and culture duration. Immortalised hepatic cell lines, such as HepG2, Hep3B, and Huh7, offer a practical alternative, supporting experimental reproducibility, and standardisation. However, their origins, mutational profiles, and patterns of hepatocyte-characteristic gene expression must be considered to ensure biological relevance.

Xenobiotic metabolism in the liver is primarily mediated by cytochrome P450 (CYP) enzymes, among which CYP3A4 is the most abundant, responsible for the oxidative metabolism of approximately 50% of all marketed drugs [2]. Because CYP3A4 activity determines the pharmacokinetics of many compounds, evaluating its induction is a critical step in drug development. The loss of or a reduction in CYP expression is associated with dedifferentiation of the hepatocyte-specific phenotype, and lower levels of these enzymes can lead to inaccurate detection of certain drug metabolism pathways [2,3].

Previous studies have shown that the expression of drug-metabolising enzyme genes is markedly reduced in HepG2 cells and in tumour samples of HCC and hepatoblastoma (HB), compared with normal hepatocytes [3,4]. Another study reported distinct differences in cytochrome P450 expression between Huh7, Hep3B, and normal hepatocytes [5]. By contrast, HepaRG cells exhibit CYP expression and activity comparable to that of primary hepatocytes (PHP) [6]. To determine whether such expression patterns are preserved in other widely used in vitro liver models, we conducted a comparative analysis of characteristic expression patterns across the following immortalised hepatic cell lines: Huh7, Hep3B, LX-2, HLF, HLE, MHCC97H, SK-Hep1, PLC/PRF/5, HepG2 and HepaRG.

To our knowledge, this is the first comprehensive analysis of DME (drug-metabolizing enzyme) genes expression in ten hepatic cell lines, achieved through the integration of public RNA-seq resources and validation against curated experimental evidence.

## 2. Materials and Methods

### 2.1. Citation Analysis of Cell Lines

The ranking of the most widely used cell lines was based on the number of articles indexed in the PubMed database as of 6 July 2025 [7]. The search was performed using the query: “CELL LINE”[Title/Abstract] AND 2015/01/01:2025/07/06[Date–Publication], where the “CELL LINE” is the name of a concrete hepatic cell line. To automate the process, we employed the Entrez module of the BioPython library (version 1.85) in the Python environment (version 3.12.2). The set of cell lines analyzed was adapted from Arzumanian et al. (2021) [8].

### 2.2. Datasets

Transcriptomic datasets were retrieved from NCBI GEO (GEO DataSets) as of 1 September 2025. The basic search query (with the experimental type filter set to “RNA-seq”) was formulated as follows: (“NAME OF CELL LINES/TISSUE”[All Fields] AND “Homo sapiens”[porgn] AND (“2015/01/01”[PDAT]:“2025/09/01”[PDAT]) AND “Expression profiling by high throughput sequencing”[Filter]).

We selected series with available pre-processed expression matrices (raw counts or TPM) that met the following inclusion criteria: (1) Organism—Homo sapiens; (2) Publication period: 2015–2025; (3) Bulk RNA-seq; (4) Availability of untreated/control samples; (5) Alignment to the hg38 assembly. We then calculated pairwise Spearman correlations within each annotated cell line and removed any samples whose mean correlation with the other samples of the same cell line was below 0.50 (Supplementary File S1). In total, 149 datasets were identified across all cell lines, PHP, and liver. The largest number of datasets was for the HepG2 cell line (32), while the smallest was for SK-Hep1 (3). An overview of dataset coverage is provided in Supplementary Table S1. Donor information for liver and PHP samples is summarized in Supplementary File S2.

### 2.3. Statistics

All computations and visualizations were performed using the R software environment (v.4.1). Gene expression data were uniformly represented as transcripts per million (TPM). When only raw counts were available, they were converted to TPM before downstream analysis using Ensembl GRCh38 version 113. To minimize potential technical variability introduced by different sequencing platforms, batch-effect correction was performed using the ComBat method implemented in the sva package, applied to log_2_-transformed data as log2(TPM+1) [9]. Values < 0.1 TPM were retained in the matrices, but treated as not expressed.

## 3. Results

### 3.1. Cell Line Authentication Challenges in Hepatic Research

**Figure 1:**
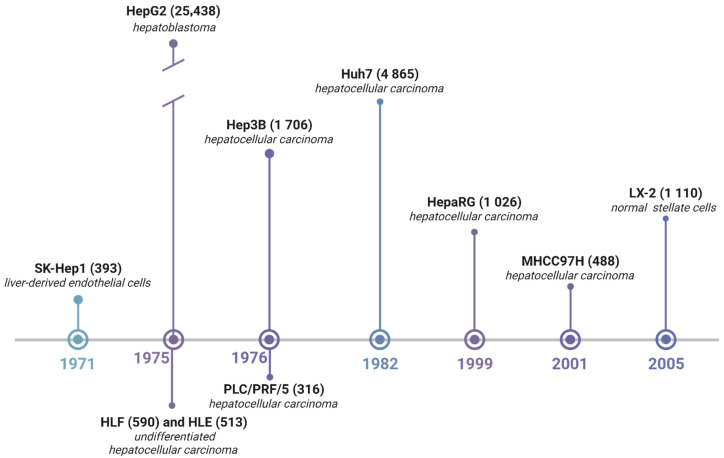
Chronology of widely used liver cell lines. Numbers in parentheses indicate the total number of publications, while bar height reflects the number of studies and thus the relative popularity of each cell line. Query to search number of articles in PubMed: “CELL LINE”[Title/Abstract] AND 2015/01/01:2025/07/06[Date—Publication].

A PubMed citation survey from 2015 to 2025 highlights a pronounced concentration of research on a few models—HepG2 (HB, 25,438 publications), Huh7 (HCC, 4865), and Hep3B (HCC, 1706)—collectively accounting for ~84% of all studies citing liver cell lines. The remaining lines—LX-2 (stellate cell, 1110), HepaRG (HCC, 1026), HLF (HCC, 590), HLE (HCC, 513), MHCC97H (HCC, 488), SK-Hep1 (adenocarcinoma, 393), and PLC/PRF/5 (HCC, 316)—each represent less than 3% of publications (Figure 1).

The history of cell line misidentification is complex, with the HepG2 cell line being a notable example. For nearly three decades, this HB line was mistakenly classified as HCC. Lopez-Terrada et al.’s landmark 2009 paper ought to have drawn a definitive line [10], classifying HepG2 as HB. Yet, perplexingly, erroneous classifications persisted, as we later documented [8,11], underscoring the inertia that can plague even well-resolved scientific controversies.

Among the earliest hepatic cell lines established, SK-Hep1 was derived in 1971 from the ascitic fluid of a patient with liver adenocarcinoma (Figure 1). Despite its widespread historical use as a hepatocyte model in numerous studies, compelling evidence now firmly establishes its endothelial origin [12]. Immunofluorescence analyses revealed definitive cytoplasmic expression of endothelial-specific markers, including eNOS, VEGF, VEGFR2, and vWF, while the canonical junctional marker CD31 was not detected [12]. In vivo transplantation studies demonstrated SK-Hep1’s capacity to form capillary-like structures in mice. Notably, CD31 expression, which is absent in vitro, was detected in these newly formed vessels, confirming their endothelial nature. Consequently, SK-Hep1 should be classified not as a hepatocyte model, but as a representative cellular model of an endothelial-like line representing the liver sinusoidal phenotype.

Like HepG2, HLE and HLF were immortalized in 1975. Both lines were derived from the hepatocellular carcinoma (HCC) of a 68-year-old male patient. The HLE culture exhibits an epithelial-like morphology, whereas HLF displays a fibroblast-like morphology. The two cultures also differ in α-fetoprotein (AFP) expression: HLE cells produced this protein up to day 187 in culture, while HLF cells did not produce it at any time examined [13]. Unlike HepG2, these lines are poorly differentiated and show high expression of GLI transcription factors, which play critical roles in intracellular signaling and serve as the principal mediators of the Hedgehog–GLI pathway [14,15]. These transcription factors are related to the Hedgehog signaling pathway, a fundamental cascade implicated in the development of multiple cancers, as well as embryonic tissue patterning and post-embryonic tissue regeneration, making them attractive targets for therapeutic intervention [16].

One year later, in 1976, the PLC/PRF/5 cell line, also known as the Alexander hepatoma cell line, was established [17,18]. The culture was derived from the liver of a 24-year-old man with HCC. The cells contain at least seven copies of the integrated hepatitis B virus (HBV) genome and secrete hepatitis B surface antigen (HBsAg). In 1976, the Hep3B line was established from liver tissue of an eight-year-old boy with HCC, and this culture also contains a 2.3-kb fragment of the HBV genome. Owing to these integrated viral sequences, both lines are widely used as models to study molecular mechanisms of virus-induced hepatocellular carcinoma. Distinguishing features of these lines include p53 status and profiles of AFP production [19]. Hep3B cells are p53-null and exhibit one of the highest levels of EGFR expression among standard HCC models [20,21]. In contrast, PLC/PRF/5 retains functional p53 and shows lower EGFR expression. The spectrum of genetic alterations in PLC/PRF/5 is biased toward cytokine-associated genes, whereas Hep3B are more often apoptosis-related [22].

Huh7 is a human HCC cell line established in 1982 from a well-differentiated tumor resected from a 57-year-old Japanese man. It exhibits a variable karyotype with complex chromosomal rearrangements, reflecting the presence of multiple clonal subpopulations [23]. Transcriptome profiling has revealed only limited similarity between Huh-7 cells and human hepatocytes [24]. Nevertheless, analysis of drug-transporter expression in Huh-7 showed mRNA levels for factors such as the farnesoid X receptor (FXR), Nrf2 (nuclear factor erythroid 2-related factor 2), and MRP2 (multidrug resistance-associated protein 2) that are comparable to—or even exceed—those in human hepatocytes [25].

HepaRG was derived in 1999 from a tumor in a female patient with chronic hepatitis C and HCC [26]. Despite this, the cells do not contain hepatitis C viral sequences. Undifferentiated HepaRG cells are bipotent hepatic progenitors that can differentiate into both cholangiocyte-like and hepatocyte-like cells under appropriate culture conditions [27]. The culture is seeded at a low density (2 × 10^4^ cells/cm^2^) and differentiated according to the supplier’s standard protocol without any selection steps, ensuring the presence of both populations within the culture [15].

Several other liver-derived lines were established after the 2000s, for example, MHCC97H in 2001 and LX-2 in 2005. MHCC97H is a subpopulation of MHCC97 (HCC, derived from a 39-year-old man) with high metastatic potential, derived from the parental MHCC97 line, which originated from a Chinese HCC patient. It has a short doubling time (~34 h) compared with the low-metastatic subpopulation MHCC97L (~60 h), has lost the Y chromosome, and is widely used to model HCC metastasis [28].

LX-2 stands out in that it was derived from healthy donors and, importantly, is not a hepatocyte model; it represents hepatic stellate cells (HSCs). LX-2 is an immortalized human HSC line. In the healthy liver, HSCs store vitamin A and regulate extracellular matrix composition and remodeling; upon liver injury, they drive fibrogenesis. LX-2 retains key features of activated HSCs: stable expression of α-SMA, vimentin, GFAP, and PDGFRβ, with secretion of procollagen, pro-MMP-2, MT1-MMP (MMP-14), TIMP-1, and TIMP-2 into the culture supernatant [29]. Morphologically, LX-2 forms elongated fibroblast-like cells at high density and displays a stellate shape at low density. With repeated passaging, LX-2 may undergo genotypic, karyotypic, and phenotypic drift, leading to subculture heterogeneity and loss of the line’s original characteristics [30]. The line is widely used to study mechanisms of hepatic fibrosis and to test anti-inflammatory agents.

Overall, hepatic cell lines exhibit a limited set of unique features that distinguish them from one another. Most share similar expression profiles of key genes, often harbour integrated viral sequences, and in many cases have closely related origins [31]. At the same time, their demand remains exceptionally high, mainly because the liver plays a central role in xenobiotic metabolism and detoxification, functions that are difficult to reproduce in vitro using other cell types. In this review, we will not attempt to cover the full spectrum of genes; instead, we will focus on those implicated in xenobiotic metabolism.

### 3.2. Drug Metabolism Genes

Genes involved in drug metabolism (DME) play a crucial role in the biotransformation of xenobiotics and endogenous compounds [32]. They encode enzymes responsible for oxidation–reduction reactions, conjugation, and metabolite transport, thereby determining the rate and direction of metabolic processes. Collectively, these mechanisms ensure detoxification, maintain homeostasis, and regulate drug pharmacokinetics [33]. Variability in DME gene expression and the presence of polymorphisms directly affect the efficacy of pharmacotherapy. Dysfunctions in these enzymes can lead to altered drug metabolism, toxic effects, or reduced therapeutic response [34].

DME genes encompass a diverse set of phase I and phase II enzymes, as well as conjugating and hydrolytic systems, which collectively determine the liver’s capacity to transform xenobiotics [35]. As with housekeeping genes, there is no single universally accepted list of DME genes. Based on a review of the literature and xenobiotic-metabolism pathway maps, we assembled a panel of 112 drug-metabolizing genes across several families (Table 1).

Within phase I enzymes, the CYP superfamily predominates, and is responsible for the oxidative biotransformation of most drugs. Genes such as *CYP3A4*, *CYP2C9*, *CYP2D6*, *CYP1A2*, and *CYP2E1* contribute disproportionately to xenobiotic clearance, while auxiliary enzymes such as PO electron transfer [36]. Additional phase I families include alcohol and aldehyde dehydrogenases (ADH/ALDH) and flavin-containing monooxygenases (FMO1–5), thereby extending substrate specificity beyond CYPs. Carboxylesterases (CESs) complement DME function by hydrolyzing ester- and amide-containing drugs, thereby generating metabolites that can under subsequent conjugation [37].

**Table 1:** List of drug-metabolizing genes.

Approved Gene Symbol	Approved Gene Name	Chromosomal Location
**Glutathione *S*-transferases (GSTs)** [38]		
*GSTA1*	Glutathione *S*-transferase (alpha) A1	6p12
*GSTA2*	Glutathione *S*-transferase A2	6p12.2
*GSTA3*	Glutathione *S*-transferase A3	6p12
*GSTA4*	Glutathione *S*-transferase A4	6p12
*GSTA5*	Glutathione *S*-transferase A5	6p12.1
*GSTK1*	Glutathione *S*-transferase kappa 1	7q34
*GSTM1*	Glutathione *S*-transferase M1	1p13.3
*GSTM2*	Glutathione *S*-transferase M2	1p13
*GSTM3*	Glutathione *S*-transferase M3	1p13.3
*GSTM4*	Glutathione *S*-transferase M4	1p13.3
*GSTM5*	Glutathione *S*-transferase M5	1p13.3
*GSTO1*	Glutathione *S*-transferase omega 1	10q25.1
*GSTO2*	Glutathione *S*-transferase omega 2	10q25.1
*GSTP1*	Glutathione *S*-transferase (pi) P1	11q13.2
*GSTT1*	Glutathione *S*-transferase theta 1	22q11.23
*GSTT2*	Glutathione *S*-transferase theta 2	22q11.2
*GSTZ1*	Glutathione *S*-transferase (zeta) Z1	14q24.3
*MGST1*	Microsomal glutathione *S*-transferase 1	12p12.3
*MGST2*	Microsomal glutathione *S*-transferase 2	4q31.1
*MGST3*	Microsomal glutathione *S*-transferase 3	1q23
*PTGES*	Prostaglandin E synthase	9q34.11
**Cytochromes P450 (CYP)** [39,40]		
*CYP1A1*	Cytochrome P450 family 1 subfamily A member 1	15q24.1
*CYP1A2*	Cytochrome P450 family 1 subfamily A member 2	15q24.1
*CYP1B1*	Cytochrome P450 family 1 subfamily B member 1	2p22.2
*CYP2A6*	Cytochrome P450 family 2 subfamily A member 6	19q13.2
*CYP2B6*	Cytochrome P450 family 2 subfamily B member 6	19q13.2
*CYP2C8*	Cytochrome P450 family 2 subfamily C member 8	10q23.33
*CYP2C9*	Cytochrome P450 family 2 subfamily C member 9	10q24.1
*CYP2C19*	Cytochrome P450 family 2 subfamily C member 19	10q23.33
*CYP2D6*	Cytochrome P450 family 2 subfamily D member 6	22q13.2
*CYP2J2*	Cytochrome P450 family 2 subfamily J member 2	1p32.1
*CYP3A4*	Cytochrome P450 family 3 subfamily A member 4	7q22.1
*CYP3A5*	Cytochrome P450 family 3 subfamily A member 5	7q22.1
*CYP2E1*	Cytochrome P450 Family 2 Subfamily E Member 1	10q26.3
*POR*	Cytochrome P450 oxidoreductase	7q11.23
**Alcohol dehydrogenases (ADH)** [41,42,43]		
*ADH1A*	Alcohol Dehydrogenase 1A (Class I), alpha subunit	4q23
*ADH1B*	Alcohol Dehydrogenase 1B (Class I), beta subunit	4q23
*ADH1C*	Alcohol Dehydrogenase 1C (Class I), gamma subunit	4q23
*ADH4*	Alcohol Dehydrogenase 4 (Class II)	4q23
*ADH5*	Alcohol Dehydrogenase 5 (Class III)	4q23
*ADH6*	Alcohol Dehydrogenase 6 (Class V)	4q23
*ADH7*	Alcohol Dehydrogenase 7 (Class IV)	4q23
*ALDH1A1*	Aldehyde dehydrogenase 1 family member A1	9q21
*ALDH1A2*	Aldehyde dehydrogenase 1 family member A2	15q21.2
*ALDH1A3*	Aldehyde dehydrogenase 1 family member A3	15q26.3
*ALDH1B1*	Aldehyde dehydrogenase 1 family member B1	9p13.1
*ALDH1L1*	Aldehyde dehydrogenase 1 family member L1	3q21.3
*ALDH1L2*	Aldehyde dehydrogenase 1 family member L2	12q23.3
*ALDH2*	Aldehyde dehydrogenase 2 family (mitochondrial)	12q24.2
*ALDH3A1*	Aldehyde dehydrogenase 3 family member A1	17p11.2
*ALDH3A2*	Aldehyde dehydrogenase 3 family member A2	17p11.2
*ALDH3B1*	Aldehyde dehydrogenase 3 family member B1	11q13.2
*ALDH3B2*	Aldehyde dehydrogenase 3 family member B2	11q13.2
*ALDH4A1*	Aldehyde dehydrogenase 4 family member A1	1p36.13
*ALDH5A1*	Aldehyde dehydrogenase 5 family member A1	6p22.3
*ALDH6A1*	Aldehyde dehydrogenase 6 family member A1	14q24.3
*ALDH7A1*	Aldehyde dehydrogenase 7 family member A1	5q31
*ALDH8A1*	Aldehyde dehydrogenase 8 family member A1	6q23.3
*ALDH9A1*	Aldehyde dehydrogenase 9 family member A1	1q24.1
*ALDH16A1*	Aldehyde dehydrogenase 16 family member A1	19q13.33
*ALDH18A1*	Aldehyde dehydrogenase 18 family member A1	10q24.1
**Carboxylesterases (CES)** [43]		
*CES1*	Carboxylesterase 1	16q12.2
*CES2*	Carboxylesterase 2	16q22.1
*CES3*	Carboxylesterase 3	16q22.1
*CES4A*	Carboxylesterase 4A	16q22.1
*CES7*	Carboxylesterase 7	16q22.1
**Flavin-containing monooxygenases (FMO)** [44]		
*FMO1*	Flavin containing monooxygenase 1	1q24.3
*FMO2*	Flavin containing monooxygenase 2	1q24.3
*FMO3*	Flavin containing monooxygenase 3	1q24.3
*FMO4*	Flavin containing monooxygenase 4	1q24.3
*FMO5*	Flavin containing monooxygenase 5	1q21.1
***N*-acetyltransferases (NATs)** [45]		
*NAT1*	*N*-acetyltransferase 1	8p22
*NAT2*	*N*-acetyltransferase 2, arylamine N-acetyltransferase	8p22
**Methyltransferases (MTs)** [34]		
*TPMT*	Thiopurine *s*-methyltransferase	6p22.3
*TMT1B*	Thiol Methyltransferase 1B	12q13.2
*COMT*	Catechol-*O*-methyltransferase	22q11.21
*HNMT*	Histamine *N*-methyltransferase	2q22.1
**Sulfotransferases (SULT)** [46,47]		
*SULT1A1*	Sulfotransferase family 1A member 1	16p11.2
*SULT1A2*	Sulfotransferase family 1A member 2	16p11.2
*SULT1A3*	Sulfotransferase family 1A member 3	16p11.2
*SULT1A4*	Sulfotransferase family 1A member 4	16p11.2
*SULT1B1*	Sulfotransferase family 1B member 1	4q13.3
*SULT1C2*	Sulfotransferase family 1C member 2	2q12.3
*SULT1C3*	Sulfotransferase family 1C member 3	2q12.3
*SULT1C4*	Sulfotransferase family 1C member 4	2q12.3
*SULT1E1*	Sulfotransferase family 1E member 1	4q13.3
*SULT2A1*	Sulfotransferase family 2A member 1	19q13.33
*SULT2B1*	Sulfotransferase family 2B member 1	19q13.33
*SULT4A1*	Sulfotransferase family 4A member 1	22q13.31
*SULT6B1*	Sulfotransferase family 6B member 1	2p22.2
**UDP-Glucuronosyltransferases (UGT)** [48]		
*UGT1A1*	UDP glucuronosyltransferase family 1 member A1	2q37.1
*UGT1A3*	UDP glucuronosyltransferase family 1 member A3	2q37.1
*UGT1A4*	UDP glucuronosyltransferase family 1 member A4	2q37.1
*UGT1A5*	UDP glucuronosyltransferase family 1 member A5	2q37.1
*UGT1A6*	UDP glucuronosyltransferase family 1 member A6	2q37.1
*UGT1A7*	UDP glucuronosyltransferase family 1 member A7	2q37.1
*UGT1A8*	UDP glucuronosyltransferase family 1 member A8	2q37.1
*UGT1A9*	UDP glucuronosyltransferase family 1 member A9	2q37.1
*UGT1A10*	UDP glucuronosyltransferase family 1 member A10	2q37.1
*UGT2A1*	UDP glucuronosyltransferase family 2 member A1	4q13.3
*UGT2A2*	UDP glucuronosyltransferase family 2 member A2	4q13.3
*UGT2A3*	UDP glucuronosyltransferase family 2 member A3	4q13.3
*UGT2B4*	UDP glucuronosyltransferase family 2 member B4	4q13.2
*UGT2B7*	UDP glucuronosyltransferase family 2 member B7	4q13.2
*UGT2B10*	UDP glucuronosyltransferase family 2 member B10	4q13.2
*UGT2B11*	UDP glucuronosyltransferase family 2 member B11	4q13.2
*UGT2B15*	UDP glucuronosyltransferase family 2 member B15	4q13.2
*UGT2B17*	UDP glucuronosyltransferase family 2 member B17	4q13.2
*UGT2B28*	UDP glucuronosyltransferase family 2 member B28	4q13.2
*UGT3A1*	UDP glycosyltransferase family 3 member A1	5p13.2
*UGT3A2*	UDP glycosyltransferase family 3 member A2	5p13.2
*UGT8* (*UGT8A1*)	UDP glycosyltransferase 8	4q26

Phase II enzymes mediate conjugation reactions that enhance solubility and excretion. These include UDP-glucuronosyltransferases (UGTs), which catalyze the glucuronidation of bilirubin, drugs, and endogenous metabolites [48]. Sulfotransferases (SULTs) and N-acetyltransferases (NAT1/2) provide additional detoxification pathways, while methyltransferases (MTs) such as *TPMT*, *COMT*, and *HNMT* regulate the metabolism of thiopurines, catecholamines, and histamine. Glutathione-S-transferases (GSTs) represent another major phase II family and play a critical role in neutralizing reactive intermediates and maintaining redox balance. Many GST genes (e.g., *GSTM1*, *GSTT1*) display copy number variation or deletions, introducing an additional layer of individual variability [49].

In clinical practice, it has long been recognized that patients respond differently to therapy due to genetic variability in DME genes [34,50]. For this reason, hepatic cell lines are widely used to evaluate potential drug toxicity and metabolic characteristics. Their application enables reproduction of key biotransformation pathways in vitro, while avoiding invasive procedures and associated ethical limitations.

Given their functional specificity, hepatic cell models are of particular importance, as the liver is responsible for the metabolism of nearly 50% of all prescribed drugs [2]. Accordingly, this review focuses on the expression of DME genes in ten of the most commonly used liver-derived cell lines that can serve as platforms for pharmacokinetic and toxicological studies.

### 3.3. Core Facts on Drug Metabolism Genes

The liver is the central organ for xenobiotic metabolism, including drugs, owing to the coordinated action of phase I and II enzymes, membrane transporters, and regulatory proteins. Comparison of hepatic cell lines with normal hepatocytes and HCC showed that most lines exhibit low expression of DME genes. In HepG2 cells, many studies have reported significantly downregulated expression of phase II GSTA family genes. For example, the key enzyme *GSTA1* is expressed roughly 11-fold lower than in primary hepatocytes and HCC tissues [51]. This enzyme conjugates a wide range of drugs and toxins, participating in the detoxification of lipid peroxidation products. Similar trends are seen in other lines: in Huh7, *GSTA1* expression is reduced 256-fold, and in Hep3B, 64-fold [31]. In SK-Hep1, it was not detected by PCR array [24].

CYP family genes are also markedly suppressed in HepG2: in several cases, expression is decreased by nearly 100,000-fold relative to primary hepatocytes and HCC [51]. These enzymes account for approximately 30–40% of the biotransformation of all clinically used drugs (including statins, immunosuppressants, macrolides, and benzodiazepines). In Huh7, *CYP2C9* levels are reduced by more than 1000-fold compared with primary hepatocytes [31]. According to Guo et al. [24], expression of the key phase I genes *CYP3A4*, *CYP2C9*, *CYP2C18*, and *CYP2C19* was not detected in SK-Hep1, HepG2, Hep3B, or Huh7 [24].

Interestingly, several phase II SULT family genes exhibit the opposite pattern—higher expression in culture than in primary hepatocytes. In particular, *SULT1A3* and SULT1A4, which catalyze the sulfation of catecholamines (dopamine, adrenaline, noradrenaline) and catechol-type drugs (levodopa), are ~4 times upregulated in HepG2 [31,51], whereas other SULT genes are downregulated. In a study that employed Western Blot for HepG2 and MHCC97H, the expression of *SULT1A3* and *SULT1A4* was increase compared to liver cells [52]. Hep3B and Huh7 also show a significant ~2-fold increase in *SULT1A3*, whereas SK-Hep1 shows no such change [24,31]. Correlation analysis of DME genes showed the strongest similarity between Huh7 and PHP (Pearson coefficient, r = 0.71) and the weakest between SK-Hep1 and PHP (r = 0.47) [24]. Notably, SK-Hep1 correlated highly with other hepatoma lines—Huh7 (r = 0.71), HepG2 (r = 0.71), and Hep3B (r = 0.72)—suggesting a shared tumor-derived program rather than a hepatocyte-like profile. The most commonly used hepatic cell line, HepG2, also differed from PHP (r = 0.60) [24].

HepaRG is considered one of the closest models to primary hepatocytes in terms of xenobiotic-metabolism gene expression. Multiple studies report that expression levels of key phase I–III enzymes in HepaRG are comparable to those in primary hepatocytes, making this line a valuable in vitro model for drugs [24,26,53,54,55].

For HepaRG cells, an organ-on-a-chip (OOC) configuration yields a transcriptome that is closer to that of primary hepatocyte than in static culture [56]. At week 4, 18 genes differed significantly, and at week 8, 15 genes; for most of these, HepaRG expression was ~2–3-fold lower than in primary hepatocytes, and rarely >5-fold (notable exceptions were *CYP1B1*, *CYP2C9*, and *CYP2C18*) [56]. The most pronounced difference was observed for CYP1B1, whose level under OOC was ~150-fold higher than in primary hepatocytes. In standard medium, 23 genes differed significantly at both 4 and 8 weeks; with HEPES buffer, 19 and 17 genes differed, respectively. Among the largest changes in standard medium were *CYP1B1* (87-fold increase), *CYP2C18* (29-fold decrease), and *SLCO1B1* (24-fold decrease). With HEPES, significant differences were observed in *CYP1B1* (79-fold increase), *CYP2C18* (67-fold decrease), *ABCB11* (29-fold decrease), and *SLCO1B1* (51-fold decrease) [56]. These data indicate that the OOC approach yields a gene-expression profile closer to physiological conditions than does static culture.

Despite LX-2, PLC/PRF/5, HLF, and HLE being among the most widely used hepatic cell models, we were unable to find publications that directly compare them with primary hepatocytes, hepatic stellate cells, or liver tissue.

### 3.4. Drug-Metabolizing Gene Expression Across Hepatic Models Using GEO Transcriptomic Datasets

To assess how DME genes (Table 1) are expressed in the ten lines we selected, we analyzed all available published and processed transcriptomic datasets (RNA sequencing). We additionally included liver tissue, primary human hepatocytes (PHP), and hepatic stellate cells. The inclusion criteria required the use of the hg38 reference genome and the availability of untreated control samples. The dataset IDs are provided in Supplementary Table S1. In total, 149 datasets were identified across all cell lines, PHP, and liver. The largest number of datasets was for the HepG2 cell line (33), while the smallest was for SK-Hep1 (3). For further analysis, we retained 108 genes and excluded *ALDH3B2*, *CES7*, *GSTT1* and *SULT6B1* genes because information for these was not available across all cell lines.

**Figure 2:**
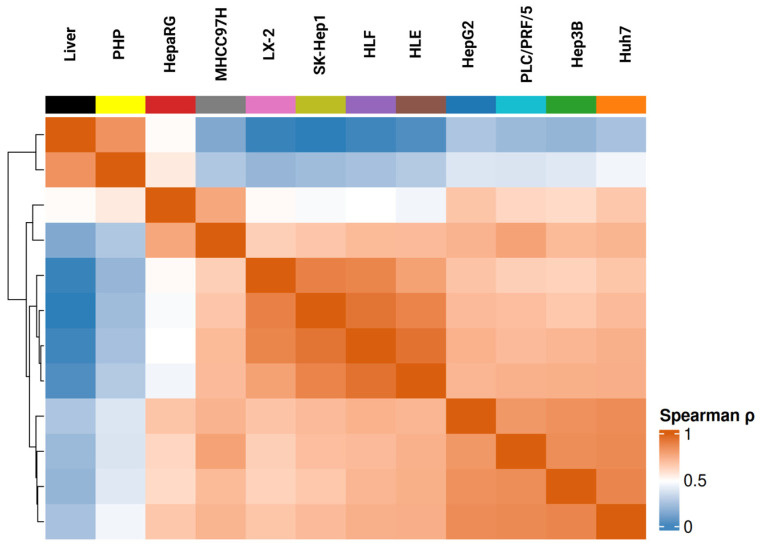
Spearman’s correlation coefficient between ten cell lines based on DME gene expression profiles, including all groups (normal liver, primary hepatocytes (PHP), and hepatic cell lines). Cell lines clustered by hierarchical clustering (average linkage). Number of datasets: normal liver—5, PHP—10, Hep3B—21, HepaRG—9, HepG2—32, HLE—9, HLF—10, Huh7—13, LX-2—9, MHCC97H—11, PLC/PRF/5—17, SK-Hep1—3.

Correlation analysis of DME gene expression revealed that PHP exhibited the highest similarity to normal liver (Spearman’s coefficient, ρ = 0.83, Figure 2). Among the cell lines, HepaRG showed the closest DME gene expression profile (ρ = 0.49), consistent with previous studies [26]. Among hepatocellular carcinoma–derived lines, HepG2 (ρ = 0.26) and Huh7 (ρ = 0.24) displayed low similarity to liver, while Hep3B (ρ = 0.19), PLC/PRF/5 (ρ = 0.21), and MHCC97H (ρ = 0.14) demonstrated divergent gene expression patterns. At the opposite end of the spectrum were HLE, HLF, LX-2, and SK-Hep1, which showed near-zero correlation with liver, reflecting their fibroblast-like origin and underscoring their limited utility in pharmacokinetic studies.

**Figure 3:**
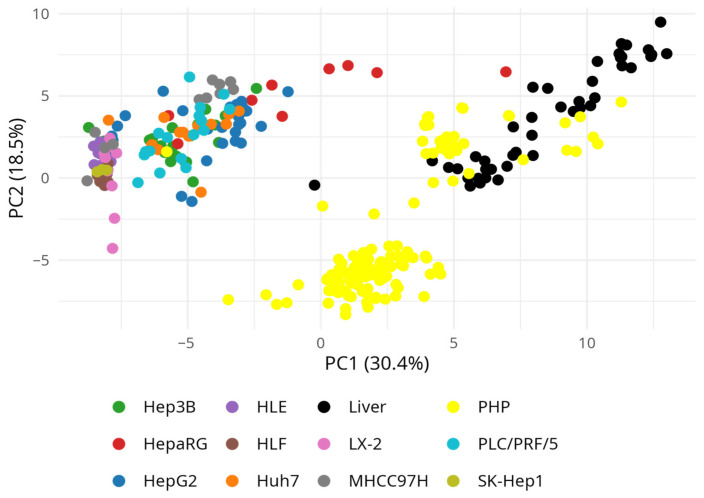
Principal component analysis (PCA) based on drug-metabolizing gene expression profiles, including all groups. Normal liver—5, primary hepatocytes (PHP)—10, Hep3B—21, HepaRG—9, HepG2—32, HLE—9, HLF—10, Huh7—13, LX-2—9, MHCC97H—11, PLC/PRF/5—17, SK-Hep1—3. The PCA is based on Euclidean distances between samples.

Principal component analysis (PCA) confirmed these findings (Figure 3). Liver, PHP, and HepaRG formed a common cluster, highlighting their preserved metabolic similarity. HepG2 and Huh7 were located near the “hepatic cluster”, retaining some hepatocyte-like features, whereas Hep3B occupied an intermediate position. PLC/PRF/5 and MHCC97H were also grouped but showed greater displacement along the principal components. In contrast, HLE, HLF, LX-2, and SK-Hep1 were positioned on the opposite side, confirming their extremely low similarity to hepatocytes.

Upon exclusion of PHP and liver, it became evident that fibroblast-derived lines (HLE, HLF, LX-2, SK-Hep1) formed a distinct, compact cluster, with within-group ρ values exceeding 0.88. HLE and HLF displayed nearly identical profiles, confirming their shared origin and functional similarity (ρ = 0.94).

In this analysis, it is also evident that HepG2 and HepaRG form distinct clusters positioned opposite to each other (ρ = 0.67) and are clearly separated from Huh7, Hep3B, PLC/PRF/5, and MHCC97H. Despite the distinctiveness of HepG2, its correlation with Huh7, Hep3B, and PLC/PRF/5 remained high (ρ = 0.84). The within-cluster correlation among Huh7, Hep3B, and PLC/PRF/5 averaged ρ = 0.85, reflecting strong similarity. MHCC97H showed moderate similarity to this cluster (ρ = 0.74), suggesting association rather than full membership. Notably, the closest partner for HepaRG was MHCC97H (ρ = 0.77), rather than any of the other hepatoma-derived lines.

Thus, correlation analysis and PCA separated the cell lines into two groups: (1) PHP-like models (HepaRG, HepG2, Huh7, Hep3B) that partially preserve the hepatic profile according to DME genes, and (2) fibroblast-like/endothelial lines (HLE, HLF, LX-2, SK-Hep1) that virtually fail to reproduce the hepatic metabolic phenotype. Although such results are expected—especially for the second group—they cannot be ignored, since these lines are commonly classified as hepatic. For example, Huh7 and HLE cells were treated with 5-azacytidine in combination with vitamin C, followed by assessments of viability, cytotoxicity, proliferation, 5hmC levels, and TET expression [57]. In another study, the antitumor activity of thymoquinone (*Nigella sativa*) was examined in SK-Hep1 cells [58], underscoring their frequent use in oncopharmacological assays despite their limited hepatocyte-like metabolic competence.

**Figure 4:**
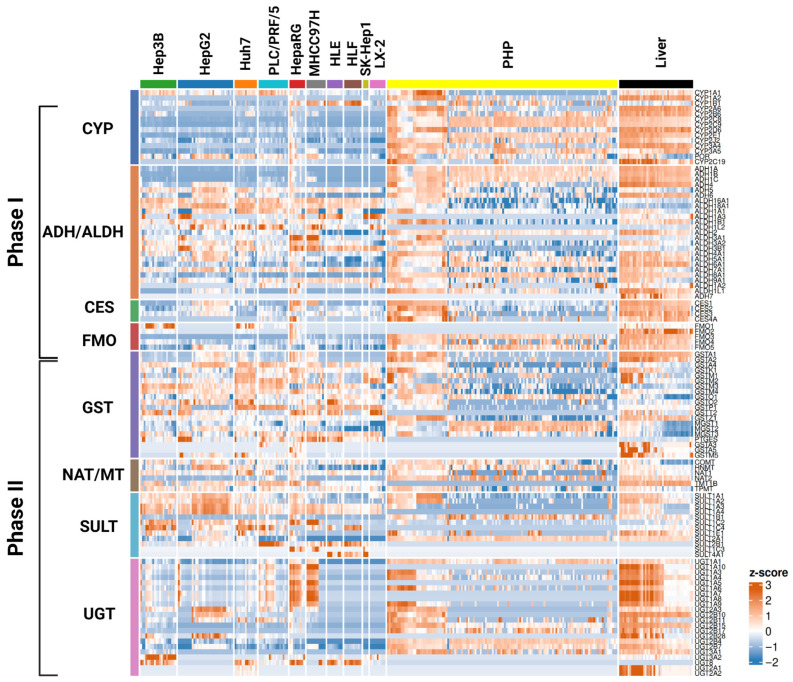
Heatmap of DME gene expression across hepatic cell lines. Each row represents an individual gene, grouped by enzyme family, and each column corresponds to a cell line. Expression values were transformed into gene-wise z-scores, enabling visualization of relative up- or downregulation across models, regardless of absolute expression levels. Number of datasets: normal liver—5, PHP—10, Hep3B—21, HepaRG—9, HepG2—32, HLE—9, HLF—10, Huh7—13, LX-2—9, MHCC97H—11, PLC/PRF/5—17, SK-Hep1—3. Samples are hierarchically clustered using complete linkage and Euclidean distance on the gene-wise z-scored DME expression profiles.

For a more detailed comparison, we generated a heatmap summarizing gene expression across all samples and cell lines (Figure 4). The most pronounced divergence between liver and cell lines was observed for cytochrome P450 genes, specifically *CYP1A2*, *CYP2A6*, *CYP2B6*, and *CYP3A4/3A5*, which showed TPM values near zero in most cell lines. Within the GST family, values comparable to those in the liver are observed for *GSTP1* in HepaRG and HepG2, and for GSTT2 in HepG2. For sulfotransferases, *SULT1A1* and MGST2 match hepatic levels in Hep3B and HepaRG, whereas in HLE/HLF, *SULT1A1* is markedly reduced. We also observed higher *SULT1A1* expression in HepG2 than in normal liver tissue.

In the UGT family, the hepatic profile is most completely recapitulated by HepaRG (including *UGT1A10*, *UGT1A3*, *UGT1A5*, *UGT1A6*, *UGT1A7*, *UGT1A8*, *UGT1A9*). *UGT2B11* is comparable to liver in HepaRG, HepG2, Hep3B, Huh7, and PLC/PRF/5, while *UGT2B28* is comparable in HepG2, Hep3B, and Huh7. A substantial subset of UGT genes in MHCC97H shows a hepatic profile, consistent with that observed in HepaRG. Notably, most SULT genes are expressed at lower levels in the liver than in cell lines, for example, *SULT1A3*, *SULT1A4*, *SULT2B1*, *SULT1C2*, and *SULT1C4*. *SULT1A3* participates in the metabolism of neurotransmitters and other compounds, such as dopamine. Its elevated expression in cell lines may bias studies involving sympathomimetics and psychoactive drugs [59].

HepaRG exhibited the most similarity to liver, for example, *ALDH1A2*, glutathione-S-transferases (*GSTA5*, *GSTM5*), carboxylesterase *CES4A*, and a broad cluster of UDP-glucuronosyltransferases of the UGT1A family were similar to liver. In several cases, individual DME genes were expressed at higher levels in cell lines than in the liver. For instance, *ALDH3A1*, a gene involved in the metabolism of chemotherapeutic drugs such as cyclophosphamide, was found to be upregulated in HepaRG [60]. Overexpression of this enzyme may result in accelerated inactivation of active cytostatic metabolites, potentially distorting assessments of drug efficacy and toxicity when using this model.

MHCC97H also reproduced hepatic levels for certain UGTs (including the UGT1A subfamily) and maintained *CES1* expression close to the liver. HepG2 retained some hepatic features, notably stable levels of individual ALDH genes (*ALDH1B1* and *ALDH3A2*) and *UGT2A3*, although its overall profile was more limited. Hep3B and Huh7 showed concordance for subsets of UGTs and SULTs, but matched liver less frequently across broader gene panels. Fibroblast-like lines (LX-2, SK-Hep1) and HLE/HLF showed the greatest deviation from the liver, consistent with their known transcriptional specificity; nevertheless, exceptions were observed for certain genes (e.g., *UGT3A2*), which reached liver-comparable levels.

HepaRG demonstrated the highest similarity across a wide DME gene panel with PHP, including *CES4A*, *CYP1B1*, *GSTA1/2*, *GSTM1*, *GSTO2*, *GSTP1*, and the *UGT1A3/1A4/1A9/1A10* cluster. HepG2 maintained levels close to PHP for *UGT2B10*, *UGT2A3*, *GSTK1*, *GSTM2*, *GSTO1/2*, *GSTP1*, and *NAT1. ALDH16A1* and *ALDH18A1* were generally higher in most lines compared to PHP, whereas *ALDH1A1* and *ALDH9A1* remained comparable across multiple models, including Hep3B, HepG2, HLE, HLF, and HepaRG. Most key CYPs (*CYP2D6*, *CYP1A2*, *CYP2A6*, *CYP2B6*, *CYP3A4/3A5*) were reduced in cell lines relative to PHP, representing the primary gap between models and primary hepatocytes. However, exceptions were noted: *CYP1A1* (Hep3B, MHCC97H, Huh7) and *CYP1B1* (HepaRG) achieved levels comparable to PHP. *FMO1* was virtually absent in both PHP and HepG2, but was expressed at liver-like levels in Hep3B, HepaRG, Huh7, and PLC/PRF/5. In contrast, several conjugation-related genes (*UGT2B4*, *PTGES*, *MGST1*, *FMO3/4/5*, *SULT1B1*) were generally lower in cell lines compared to PHP, whereas others (*UGT8*, *UGT3A2*, *UGT2B28*, *UGT2B7*, as well as *UGT1A1/1A5/1A7/1A8/1A10* and *SULT2B1*, *SULT1C4/1C2/1A4/1A3/1A2*) were upregulated in several models. Hep3B showed a high degree of concordance with PHP (e.g., *SULT1A2*, *SULT1E1*), whereas TPMT remained consistently expressed at comparable levels across all models.

Taken together, HepaRG ranked first in terms of similarity to both liver and PHP, most accurately reproducing DME gene expression patterns. HepG2, Hep3B, and Huh7 formed a cluster retaining partial hepatic characteristics. The remaining lines showed more pronounced deviations from the reference profile and are therefore better suited for specialized applications—such as studies of fibrosis, oncogenic processes, or cell–cell interactions.

## 4. Discussion

Analysis of public datasets and the literature confirms a previously noted publication bias toward the HepG2, Huh7, and Hep3B cell lines, which together account for ~84% of studies citing hepatic cell lines (Figure 1). Their popularity is largely driven by their relative functional proximity to primary hepatocytes. However, focusing on only these three models reduces the likelihood of detecting lineage-specific features that may emerge in less frequently used lines. For example, HepaRG more faithfully reproduces CYP induction, whereas lines with low *CYP3A4* expression fail to reflect actual hepatic metabolism, leading to underestimation of clinically meaningful drug–drug interaction (DDI) risk. Consequently, when the overwhelming majority of data are derived solely from HepG2, Huh7, and Hep3B cells, the results predominantly describe properties of these models rather than hepatic processes. These genes are critical when using hepatic cell lines in pharmacological and toxicological research. Moreover, according to the ICH M12 (2024) guideline [61], assessment of DDI risk should be performed in PHP from at least three individual donors, quantifying induction at the mRNA level. It should always include *CYP3A4*, *CYP2B6*, and *CYP1A2* as markers of the PXR/CAR/AhR pathways.

In addition to a systematic literature review on DME gene expression, we analyzed available transcriptomic expression matrices for ten widely used hepatic cell lines, benchmarking them against normal liver and PHP to identify shared and specific profiles. To this end, we not only retrieved datasets for the cell lines but also included PHP and normal liver as reference groups. In total, we processed 147 datasets, providing broad coverage across ten hepatic cell lines, normal liver, and PHP (on average, 13 datasets per cell line).

Convergence of HepaRG with PHP and liver is supported by both the literature and by our data [24,26,53,54,55], where we observed a Spearman’s ρ of 0.90 for PHP–HepaRG and 0.67 for liver–HepaRG, higher than for other lines. As expected, culture conditions are critical: organ-on-a-chip (OOC) configurations shift the HepaRG transcriptome closer to physiological conditions than static culture, although several genes (e.g., *CYP1B1*) exhibit pronounced deviations [56]. The practical takeaway is clear: for ADME (Absorption, Distribution, Metabolism, Excretion), toxicology, and drug-metabolism applications, HepaRG is a rational first-choice platform, particularly in OOC formats.

A family-specific analysis reveals that CYP genes are the primary drivers of the “gap” between cell lines and liver: for *CYP1A2*, *CYP2A6*, *CYP2B6*, and *CYP3A4/CYP3A5*, TPM values are near zero in most lines, with rare exceptions (*CYP1A1* and *CYP1B1* in certain models). This aligns with reports of multi-log reductions in CYP expression in HepG2, with CYP3A4 levels to 130,000 times lower [51]. Conjugation families show a more nuanced picture: MGST1 is often comparable to liver; HepaRG reproduces the UGT1A cluster best (*UGT1A10/1A3/1A5/1A6/1A7/1A8/1A9*), *UGT2B11* approaches liver levels in several lines (HepaRG, HepG2, Hep3B, Huh7, PLC/PRF/5), and *UGT2B28* is preserved in HepG2/Hep3B/Huh7. Notably, MHCC97H shows UGT expression profiles that are also similar to those of liver, consistent with its increasing use as a model in drug discovery studies [62].

Among GSTs, some isoforms (e.g., *GSTP1* in HepaRG and HepG2, *GSTT2* in HepG2) retain liver-like levels of expression. For SULTs, bidirectional shifts are observed: *SULT1A1* is reduced in HLE/HLF, whereas *SULT1A3/1A4/2B1/1C2/1C4* can exceed liver levels in culture; in HepG2, Hep3B, and Huh7, *SULT1A3* is frequently elevated (~2–4×) [31,51,52], which is critical when modeling catechol substrates. For carboxylesterases, *CES4A* levels in HepaRG cells often do not differ from those in the liver. Taken together, this explains why HepaRG exhibits the greatest concordance with PHP across a broad DME panel, whereas HepG2, Hep3B, and Huh7 form a separate cluster with partial matches, and fibroblast-like, endothelial, and non-hepatocytic lines systematically deviate from the reference.

We did not observe systematic differences in the activation of Phase I and Phase II genes. The main exceptions were several SULT genes and HNMT (phase II), which were more strongly upregulated in HepG2 than in liver tissue or PHP. Additionally, some ALDH genes (phase I), such as *ALDH3A1/3B1/16A1/1L2*, and *SULT1C2* (phase II), were more upregulated in MHCC97H than in liver and PHP. So, cell lines could not be cleanly partitioned into high- versus low-expression groups across DME phase I and phase II pathways.

From a practical standpoint, the choice of cell model should be driven by the research aim. For ADME and toxicology, HepaRG is the preferred line, especially in organ-on-a-chip or other microfluidic systems. For transporter and regulatory-axis studies, HepG2, Huh7, or MHCC97H can be used, primarily for targeted validation. For HBV-related phenotypes, a combination of PLC/PRF/5 and Hep3B is reasonable, considering the differences in p53 and EGFR status.

The LX-2 and SK-Hep1 lines are best applied according to their intended purpose—for modeling fibrosis and endothelial phenotypes, respectively—while avoiding generalizations in the context of ADME. Similarly, HLE and HLF show weak correlation with liver and PHP and, according to clustering analysis, align more closely with LX-2 and SK-Hep1 than with HepG2, Huh7, Hep3B, PLC/PRF/5, and MHCC97H. Therefore, these lines should likewise be restricted to specialized applications (fibrosis, endothelium) rather than being extrapolated to pharmacokinetics in general.

Because native hepatic models often show incomplete expression of key CYPs and conjugating enzymes, engineered derivatives have been developed to restore specific metabolic functions. Such lines are engineered to stably express specific cytochrome P450 enzymes that are otherwise underrepresented in conventional hepatic models. For example, HepaRG-CYP2D6 cells express *CYP2D6* under a constitutive promoter, thereby restoring functional activity toward typical *CYP2D6* substrates. Similarly, HepG2-CYP2E1 cells, generated via pREP9-CYP2E1 transfection and G418 selection, exhibit enhanced metabolism of ethanol and small xenobiotics via CYP2E1 [63]. More complex constructs include the multi-CYP HepG2 model, in which four major CYP genes (*CYP2C9*, *CYP2C19*, *CYP3A4* and *POR*) are introduced using a mammalian artificial chromosome, yielding a stable, physiologically relevant tool for pharmacokinetic and toxicological assessment [64,65]. Recently, HuH-7-CYP3A5 cells created via CRISPR/Cas9-mediated knock-in demonstrated selective restoration of *CYP3A5* activity and response to PXR/CAR agonists [66].

ICH M12 requires that drug–drug interaction (DDI) studies use physiologically relevant in vitro systems that truly reflect the human liver. Still, widely used hepatic cell models differ strongly in the expression of CYP enzymes, conjugating enzymes, and transporters that drive DDIs. By systematically mapping these differences, our study provides a molecular framework with which to qualify and select the most appropriate cell models for ICH M12–compliant DDI testing, improving translational confidence in human risk prediction.

Our research integrated multiple transcriptomic datasets generated by different research groups. In contrast to previous single-experiment analyses, we provide an integrated overview of gene expression across PHP, liver, and hepatic cell lines. Despite the comprehensive nature of our study, its limitations must be acknowledged. We relied not on proprietary data or even raw datasets, but on pre-processed expression matrices available from public resources. Batch-effect correction was performed only with respect to the sequencing platform, as the application of additional criteria might have masked genuine biological variation. Consequently, this study is primarily descriptive and aims to systematize existing knowledge of the behavior of key DME genes across different hepatic cell models. We hope that the results will be helpful to researchers in selecting the most appropriate cell line for specific experimental objectives.

## 5. Conclusions

The analysis demonstrated distinct differences among widely used hepatic cell models, normal liver tissue, and PHP in drug metabolism. PCA revealed a consistent trend: HepaRG most closely reproduced the hepatic phenotype; HepG2, Huh7, Hep3B, PLC/PRF/5, and MHCC97H retained only partial hepatocyte-like features; and HLE, HLF, LX-2, and SK-Hep1 clustered together as fibroblast-like/endothelial models, consistent with their origin, and are therefore poorly suited for pharmacokinetic and metabolic studies.

In conclusion, no single cell line fully substitutes for liver function. Each model exhibits inherent limitations and biases in the expression profile of DME genes, which must be considered when selecting an appropriate system for specific research applications.

## Data Availability

No new data were created or analyzed in this study. Data sharing is not applicable to this article.

## Supplementary Materials

The following supporting information can be downloaded at: https://www.mdpi.com/article/10.3390/biomedicines13112722/s1, Table S1: Dataset IDs for each cell line, normal liver, and primary hepatocytes. File S1: Correlation analysis results across datasets for each cell line. File S2: Donor metadata for liver tissue and PHP samples.

## Abbreviations

CYP	Cytochrome P450
PHP	Primary Hepatocytes
HCC	Hepatocellular Carcinoma
HB	Hepatoblastoma
AFP	Alpha-fetoprotein
HBV	Hepatitis B Virus
HBsAg	Hepatitis B Surface Antigen
HSC	Hepatic Stellate Cell
DME	Drug-metabolizing enzymes
ADH/ALDH	Alcohol and Aldehyde Dehydrogenase
FMO	Flavin-containing Monooxygenase
CES	Carboxylesterase
UGT	UDP-glucuronosyltransferase
SULT	Sulfotransferase
NAT	N-acetyltransferase
MT	Methyltransferase
GST	Glutathione-S-transferases
PCA	Principal Component Analysis
OOC	Organ-On-A-Chip
