# Supplementary material for: Drug-Metabolizing Gene Expression Identity: Comparison Across Liver Tissues and Model Cell Lines
Source: Biomedicines. 2025 Nov 6;13(11):2722. doi: 10.3390/biomedicines13112722 (PMC12650171; doi:10.3390/biomedicines13112722)
Supplement: Supplementary file 1 [file biomedicines-13-02722-s001.zip › Supplementary File S2.pdf]

Donor metadata for liver tissue and primary human hepatocyte (PHP) samples used in the study

PMID	GEO	Group / Dataset	n	Male/Female	Age, years (mean ± SD)
**Liver**					
30653341	GSE126848	Normal-weight controls	14	14 / 0	39.5 ± 12.0
34336890	GSE171294	Control	10	5 / 5	51.4 ± 10.6
**Primary Hepatocytes**					
32724080	GSE139896	Healthy; no drugs/tobacco/alcohol	3	2/1	24 – 51 (51, 24, 30 years)
